# Effect of Feeding Level on Growth and Slaughter Performance, and Allometric Growth of Tissues and Organs in Female Growing Saanen Dairy Goats

**DOI:** 10.3390/ani14050730

**Published:** 2024-02-27

**Authors:** Juan Huang, Shuai Jiao, Yuze Fu, Wei Zhao, Qiyu Diao, Tao Ma, Naifeng Zhang

**Affiliations:** Key Laboratory of Feed Biotechnology of the Ministry of Agriculture and Rural Affairs, Institute of Feed Research of Chinese Academy of Agricultural Sciences, Beijing 100193, China; 82101211214@caas.cn (J.H.); 1635818839@163.cn (S.J.); 771230044@163.cn (Y.F.); zhaowei45@nwafu.edu.cn (W.Z.); diaoqiyu@mail.caas.net.cn (Q.D.); matao@caas.cn (T.M.)

**Keywords:** Saanen dairy goats, growth performance, slaughter performance, organ index, allometric growth

## Abstract

**Simple Summary:**

With the rapid expansion of large-scale dairy goat farming in China, Saanen goats have become the preferred choice for farmers due to their strong reproductive capacity and high milk production. However, there is a lack of information on the growth and development patterns of the body and visceral organs. To our knowledge, this is the first report documenting the allometric growth of tissues and organs in Saanen dairy goats. This study not only explores developmental trajectories of dairy goats at this growing stage, helping to optimize feeding management strategies and feed formulation, but also provides a fundamental benchmark for developing comprehensive nutrient requirements for dairy goats.

**Abstract:**

This study aimed to investigate the effect of feeding level on the growth and slaughter performance, and allometric growth of tissues and organs in female growing dairy goats. The trial included 10–20 and 20–30 kg weight stages with 48 female goat kids. The 24 goat kids in each stage were divided into 8 blocks based on weight, with 3 kids per block. Then, three kids from each block were randomly assigned to one of the three treatments, namely ad libitum (AL100), 70% of ad libitum (AL70), or 40% of ad libitum (AL40). The slaughter trial was conducted when the AL100 kids reached the target weight of 20 or 30 kg. The results showed that the ADG and feed conversion rate showed a linear decline as the feed level decreased (*p* < 0.05). Compared with the AL70 and AL100 groups, the AL40 group exhibited lower shrunk body weight, empty body weight, hot carcass weight, net meat rate, carcass meat rate, and visceral fat weight (*p* < 0.05) in both stages. Moreover, the AL40 group showed lower weights for skin and mohair, blood, rumen, small intestine, large intestine, mammary gland, and uterus than the AL70 and AL100 groups (*p* < 0.05) in both stages. However, feeding level did not affect organ indices in the two stages (*p* > 0.05). The bone, skin and mohair were isometric (b ≈ 1), but the muscle, visceral fat, and most internal organs were positive (b > 1) in both stages. In conclusion, feeding level affects the growth and development of dairy goats, which vary depending on the body weight stage and specific tissues and organs.

## 1. Introduction

Since the 1960s, the global goat population has been steadily increasing due to shifting economic patterns and the evolving dietary preferences of humanity. By 2021, the global goat population exceeded one billion, marking an increase of over 46% since 2000 [1]. Asia accounts for over 50% of the world’s goat population, with China, India, and Pakistan having the largest numbers of these creatures [2,3]. With the rapid expansion of large-scale dairy goat farming, Saanen dairy goats have become the favored choice of breeders due to their robust reproductive capabilities and high milk yields. In recent years, progress has been made in research on the growth, slaughter, and organ development of dairy goats. A balanced nutritional feed has been found to promote the growth and development of dairy goats, improving their milk production performance during lactation [4]. Adequate protein intake also enhances their growth rate and muscle development [5,6]. Research on organ development in dairy goats has primarily focused on the mammary gland and digestive system [7]. Proper diet and high-nutrition feed can promote the development of the mammary gland, resulting in increased milk production and improved milk quality [6]. Furthermore, the development of the digestive system plays a crucial role in the food digestion, absorption, and growth of dairy goats [8]. In terms of slaughter, researchers are dedicated to optimizing slaughter techniques and introducing new slaughter equipment such as electronic anesthesia and precise-positioning slaughter technologies [9]. These advancements aim to reduce the stress and pain experienced by dairy goats during the slaughter process, while improving efficiency and safety [10]. Nevertheless, there exists a dearth of data supporting the nutritional maintenance, growth requirements, growth performance, and feed utilization efficiency of dairy goats across diverse physiological stages; specifically information regarding the growth and developmental patterns of the body and internal organs remains conspicuously absent. Hence, the principal objective encompassed an exhaustive examination of the repercussions arising from varied feeding levels on the growth performance, slaughter attributes, tissue and organ maturation, and the distinctive patterns of allometric growth manifest in Saanen dairy goats. This study garnered insights into the nutritional prerequisites and developmental trajectories of dairy goats in this physiological phase, providing a fundamental benchmark for developing comprehensive feeding standards tailored to the unique needs of dairy goats.

## 2. Materials and Methods

This study was conducted at the Inner Mongolia Academy of Agricultural & Animal Husbandry Science Experimental Base of Togtoh Prefecture, located at coordinates 111°2′30″ E, 40°35′15″ N, at an altitude of 1000 m. All research and experimental procedures were approved by the CAAS Animal Ethics Committee, and humane animal care and procedures were strictly followed throughout the experiment, as specified by protocol number IFR-CAAS-20220515. Throughout the experiment, the minimum and maximum temperatures recorded were 22.76 °C and 29.96 °C, and the minimum and maximum relative humidity in the air were 36.98% and 48.77%, respectively.

### 2.1. Animals, Management, and Experimental Design

The trial included two liveweight stages of 10–20 and 20–30 kg, with 24 female growing goat kids in each stage, totaling 48 kids. With a randomized block design, the 24 goat kids in each stage were divided into 8 blocks based on live weight, with 3 kids per block. Then, three kids from the same block were randomly assigned to one of the three feeding levels, namely ad libitum (AL100), 70% ad libitum (AL70), or 40% ad libitum (AL40). The feeding levels were determined based on pre-trial feed intake, and the AL40% group was designed to meet the maintenance requirements of energy and protein. During the trial, the goat kids were fed thricely at 0800, 1400, and 1800, and drank freely.

Animals in both stages received diets (Table 1) formulated according to the recommendations of the National Research Council (NRC) (2007) for growing dairy goats, with an average body weight of 15 kg, dry matter intake (DMI) of 0.57 kg/d, average daily gain (ADG) of 150 g/d, and an average body weight of 25 kg, DMI of 0.94 kg/d, and ADG of 150 g/d. The corresponding metabolizable energy (ME) and metabolizable protein (MP) of diets for dairy goats at these two weight stages were 7.56 MJ/d (1.80 Mcal/d), 67 g/d, and 9.49 MJ/d (2.26 Mcal/d), 78 g/d.

### 2.2. Growth Performance

During the trial, both the feed offered and refused were weighed daily to calculate the DMI. The goat kids were weighed at the beginning and end of the experiment, respectively, for the calculation of initial body weight (IBW), final body weight (FBW), and ADG. The Feed-to-Gain ratio (F/G) was quantified as the ratio of DMI to ADG [12].

### 2.3. Slaughter Trials

As each dairy goat in AL100 attained the target liveweight of 20 or 30 kg, three dairy goats within the same block were concurrently slaughtered until all 48 animals underwent processing. Prior to slaughter, goats were fasted for 16 h from the feed with free access to water. Then, the animals were slaughtered according to the standard commercial procedures. The Shrunk Body weight (SBW) was determined by weighing just before slaughter [13]. The blood was weighed during the slaughter process. After slaughter, the empty body weight (EBW) was determined by removing the gastrointestinal tract (GIT), bladder, and gall bladder contents. Then the head, feet, skin, mohair, all the viscera, and internal fat depots were weighed and removed to determine the hot carcass weight (HCW) [14]. The Net meat weight (NMW) was obtained by removing the bones from HCW. The dressing percentage (DP), net meat percentage (NMP), and carcass meat rate (CMR) were obtained as the proportion of HCW to SBW (DP = 100 × CW/SBW), NMW to SBW (NMP = 100 × NMW/SBW), or NMW to HCW (CMR = 100 × NMW/HCW) as defined by Atsbha et al. (2021) and He et al. (2023) [15,16]. After emptying and flushing the digestive tract, the weights of the rumen, Omasum, abomasum, reticulum, small intestine, and large intestine were recorded. The visceral organ index was calculated as the proportion of organ weight to SBW.

### 2.4. Statistical Analysis

The raw data were collected using Excel 2010. The data were analyzed using a Randomized Block Design of variance in SPSS software, Version 27.0. Effects were deemed significant when the *p*-value was below 0.05 (*p* < 0.05). Duncan’s method was taken for multiple comparisons when the analysis of variance results were significant.

After processing the data, a Huxley’s allometric equation model was used to fit the intensity of growth and development of the various internal organs of the goats [17]. Huxley’s allometric equations are commonly used to study the relationship between variables related to different parts of the body and are generally fitted by an exponential equation with Y = a × X^b^ for heterogeneous growth. X is the independent variable (SBW), Y is the dependent variable (muscle, fat, bone, skin, blood, viscera, organ weight, etc.), a is the allometric growth constant, and b is the allometric growth slope, with b = 1 indicating that the growth of the differentiated study subject remains consistent with the whole (isometry). When b < 1, it means that part of the study subject grows slower than the whole (negative allometry); when b > 1, it means that part of the study subject grows faster than the whole (positive allometry). The significance of differences in the slope of the linear regression was assessed using SPSS software version 27.0. To perform the analysis, navigate to Analyze -> General Linear Model -> Univariate. Effects were deemed significant when the *p*-value was below 0.05 (*p* < 0.05).

## 3. Results

### 3.1. Growth Performance

The IBW did not show differences (*p* > 0.05) among groups in both weight stages (Table 2), indicating successful blocking. However, significant differences (*p* < 0.05) were observed in FBW, ADG, DMI, and F/G with the decrease in feed levels in both stages. In the 10–20 kg weight stage, the DMI decreased in a quadratic (*p* < 0.05) manner as the feed level decreased. Correspondingly, the ADG and FBW showed a quadratic decline (*p* < 0.05) with the feeding restriction, whereas F/G showed a quadratic enhancement (*p* < 0.05). The DMI, ADG, and FBW of goats were lower (*p* < 0.05) in the AL40 group than in the AL100 and AL70 groups, whereas the F/G in the AL40 group was greater (*p* < 0.05). However, the ADG and FBW showed a linear decline (*p* < 0.05), whereas F/G showed a linear enhancement (*p* < 0.05) with the decreased feed level in the 20–30 kg weight stage, as a result of the DMI linear decline. The DMI, ADG, and FBW of goats in the AL40 and AL70 groups were lower (*p* < 0.05) than in the AL100 group. Furthermore, lower DMI, ADG, and FBW were observed in the AL40 group compared with the AL70 and AL100 groups (*p* < 0.05), contributing to a high F/G in the AL40 group compared with AL70 and AL100 group (*p* < 0.05).

### 3.2. Slaughter Performance

Significant differences (*p* < 0.05) were observed in SBW, EBW, HCW, net meat weight, net meat percentage, carcass meat rate, and visceral fat weight with the decrease in feed levels in both stages (Table 3). However, the dressing percentage and visceral fat percentage were not different among the groups. In the 10–20 kg weight stage, the SBW, EBW, HCW, net meat percentage, bone weight, ratio of meat to bone, and visceral fat weight showed a quadratic decline (*p* < 0.05) with the feeding restriction, whereas net meat weight and carcass meat rate showed a linear decline (*p* < 0.05). The SBW, EBW, HCW, net meat weight, net meat percentage, carcass meat rate, bone weight, ratio of meat to bone, and visceral fat weight of goats were lower (*p* < 0.05) in the AL40 group than in the AL100 and AL70 groups. Similarly, the SBW, EBW, HCW, net meat weight, net meat percentage, carcass meat rate, bone weight, ratio of meat to bone, and visceral fat weight showed a linear decline (*p* < 0.05) with the decreased feed level in the 20–30 kg weight stage. The SBW, EBW, HCW, net meat weight, and visceral fat weight of goats in the AL40 and AL70 groups were lower (*p* < 0.05) than in the AL100 group. Furthermore, lower SBW, EBW, HCW, net meat weight, net meat percentage, carcass meat rate, and visceral fat weight were observed in the AL40 group compared with the AL70 and AL100 groups (*p* < 0.05).

### 3.3. Stomach Development

During both weight stages, it was evident that the rumen was the heaviest among all stomach chambers and accounted for the highest proportion of the total stomach weight (Table 4). at the 10–20 kg stage of goats, the weights of the rumen, total stomach, and the weight proportion of rumen to total stomach or SBW quadratic declined (*p* < 0.05), but the weights of reticulum, Omasum, and abomasum linearly decreased, with the feeding level falling (*p* < 0.05). The Rumen, Reticulum, Omasum, Abomasum, Total stomach, and the weight proportion of rumen to the total stomach or SBW of goats were lower (*p* < 0.05) in the AL40 group than in the AL100 and AL70 groups.

During the 20–30 kg stage, the weights of rumen, reticulum, total stomach, and the weight proportion of rumen to total stomach or SBW and abomasum to total stomach or SBW linearly decreased, with the feeding level falling (*p* < 0.05); however, the weights of the Omasum, and the proportion of Omasum to SBW quadratically declined (*p* < 0.05). The weights of the rumen, Omasum, and total stomach, of goats were lower (*p* < 0.05) in the AL40 and AL70 groups than in the AL100 group. Furthermore, lower weights of rumen and total stomach were observed in the AL40 group compared with the AL70 and AL100 groups (*p* < 0.05).

### 3.4. Tissue and Organ Development

At the 10–20 kg stage, the weights of the heart and liver quadratically declined (*p* < 0.05; Table 5), but the weights of the spleen, lung, kidney, and liver index linearly decreased with the feeding level falling (*p* < 0.05). The weights of the heart, liver, spleen, lung, and kidney of goats were lower (*p* < 0.05) in the AL40 group than in the AL100 and AL70 group. Furthermore, lower weights of lungs and kidneys were observed in the AL40 and AL70 groups compared with the AL100 group (*p* < 0.05). At the 20–30 kg stage, the weights of the heart, liver, spleen, lung, kidney, and liver index linearly declined (*p* < 0.05), but the heart index quadratically decreased with the feeding level falling (*p* < 0.05). The weights of the heart, liver, and spleen of goats were lower (*p* < 0.05) in the AL40 group than in the AL100 and AL70 group. Furthermore, lower heart, liver, and spleen weights were observed in the AL40 and AL70 groups compared with the AL100 group (*p* < 0.05). However, feeding levels did not affect organ indices in 10–20 kg and 20–30 kg dairy goats (*p* > 0.05).

At the 10–20 kg stage, the weights of the head, hooves, skin and mohair, blood, small intestine, large intestine, uterus, and mammary gland linearly declined (*p* < 0.05; Table 6), and the weights of the head, skin and mohair, blood, and mammary gland quadratically decreased, with the feeding level falling (*p* < 0.05). The weight of the head, hooves, skin and mohair, blood, small intestine, large intestine, uterus, and mammary gland of the goats were lower (*p* < 0.05) in the AL40 group than in the AL100 and AL70 groups. Furthermore, lower weights of blood and large intestine were observed in the AL40 and AL70 groups compared with the AL100 group (*p* < 0.05). At the 20–30 kg stage, the weights of skin and mohair, blood, small intestine, large intestine, and uterus linearly declined (*p* < 0.05), and the weight of skin and mohair quadratically decreased, with the feeding level falling (*p* < 0.05). The weight of skin and wool, blood and small intestine of goats were lower (*p* < 0.05) in the AL40 group than in the AL100 and AL70 group. Furthermore, lower weights of blood and small intestine were observed in the AL40 and AL70 groups compared with the AL100 group (*p* < 0.05). However, feeding levels did not affect organ indices in 10–20 kg and 20–30 kg dairy goats (*p* > 0.05).

### 3.5. Allometric Growth of Tissues and Visceral Organs

The Huxley’s allometric growth equation is a mathematical model used to describe the situation where different parts the animal [17], such as the muscles and bones, grow at different rates during the growth process [18]. As there were no significant differences in the allometric growth coefficients of all the tissue and visceral organs among the three feeding levels, the exponential equations were fitted with all values of the three feeding levels. The fitted equations obtained by fitting the dairy goat organs and tissues to SBW showed that the R-squared of the regression curves was higher, and the *p* values were lower than 0.05 (Table 7), indicating that the equations can accurately describe the development manner of the dairy goat organs and tissues. The order of tissue development at 10–20 kg and 20–30 kg stages were visceral fat > muscle > skin and mohair > bone, and visceral fat > muscle > bone > skin and mohair, respectively (Table 7). The muscle and visceral fat allometric coefficients were positive (b > 1, *p* < 0.05) at the 10–20 kg and 20–30 kg stages, indicating late maturing. Whereas the allometric coefficient for bone and skin and mohair was isometric (b≈1) and lower than muscle and visceral fat (*p* < 0.05).

For the Huxley’s allometric growth of internal organs (Table 8), the allometric coefficients for most organs were positive (b > 1, *p* < 0.05) at the 10–20 kg and 20–30 kg stages, indicating later maturing, except for abomasum at the 20–30 kg stage, which was negative (b < 1, *p* < 0.05) and indicating early maturing. The mammary gland in the 10–20 kg stage and the small intestine in the 20–30 kg stage were positive (b > 1, *p* < 0.05) and highest, indicating later maturing.

## 4. Discussion

### 4.1. Growth Performance

The basal diet in this study was formulated following the NRC (2007) requirements for female dairy goats [11]. In this study, a total mixed ration was employed to prevent any feed selection bias caused by ad libitum feeding, which could lead to varying proportions of ingredients consumed compared to animals fed restrictively [19]. The results indicated that the goats in the AL100 group achieved an actual ADG of 154.31 and 144.53 g/d at the 10–20 and 20–30 kg stages, respectively, which were close to the NRC’s target ADG of 150.0 g/d [11]. However, Our DMI exceeds the NRC recommendation by 35% and 6.5% at the 10–20 kg and 20–30 kg stages, which were 0.57 and 0.94 kg for dairy goats in this weight range to achieve the same ADG. Furthermore, the DMIs in this study, which were 5.1% and 4.0% of BW for 10–20 kg and 20–30 kg dairy goats, yielding a mean value of 4.5%, were higher than the AFRC (1998) findings, that were 4% of BW for the DMI of 20–35 kg dairy goats, as [20] was also higher than the NRC guidelines, whose estimated DMI for 10–20 and 20–30 kg dairy goats is 3.79% and 3.75% of BW. Teixeira et al. argued that the equation proposed by AFRC underestimates DMI [21,22]. These variations can be attributed to various factors. Firstly, the forage materials, both in terms of type and quality, which vary due to regional and husbandry practices, significantly influence the foraging behavior and developmental trajectory of Saanen dairy goats [23,24]. This urges us to conduct additional research to assess the nutritional value of our feed resources, ultimately improving the dietary formulation for dairy goats and enhancing their performance [25]. Secondly, prolonged periods of selective breeding in different regions have resulted in distinct Saanen goat strains, leading to differences in their growth tendencies [26,27]. Additionally, atmospheric parameters, including temperature, humidity, and altitude, exert imperative influences on the appetitive inclinations and growth kinetics of Saanen goats [28,29]. Comprehending the growth and development of goats and the factors influencing these processes is crucial due to its impact on production efficiency and product quality. These findings emphasize the necessity and urgency of developing precise nutritional requirements for Chinese dairy goats.

### 4.2. Slaughter Performance

Slaughter performance, including carcass weight and dressing percentage, directly manifests the growth performance and economic value of animals [5,15]. Differences among treatments in SBW, EBW, HCW, net meat percentage, the ratio of meat to bone, and visceral fat percentage were observed at both stages in this study. The lower HCW and other slaughter performance recorded in the AL40 groups reflected that restricted feed consumption reduced muscle growth and fat deposition [15,30]. In this study, the body composition of goats undergoes substantial changes with age. In the 20–30 kg stage, dairy goats exhibit notable muscle and visceral fat deposition compared with that of the 10–20 kg stage. The Saanen goat, in common with other dairy goat breeds, tends to store a greater proportion of its fat internally, rather than at subcutaneous or intermuscular sites [31]. It has been observed that as the animal’s EBW increases and approaches physiological maturity, the deposition of muscle tissue decreases while the deposition of fat increases. This pattern is considered a normal growth pattern in animals, where fat tissue shows later growth compared to overall body growth [14,32]. Tissue development in both weight ranges reveals that feeding levels influence the rate of tissue development but do not alter the location of tissue deposition. The development of different tissues follows a specific sequence, starting with bone, followed by muscle and adipose tissue [32]. This implies that animals fed ad libitum typically develop all tissues, while underfed animals may experience compromised development [33]. This observation is supported by the existing literature [34], suggesting that older goats primarily store high-calorie fat internally during weight gain, while younger goats rely on the growth of muscles, bones, and various organs to increase body weight. However, there was no difference in the dressing percentage of dairy goats at either weight stage, indicating that the dressing percentage of Saanen dairy goats remained relatively stable in general.

### 4.3. Tissue and Organ Development

A well-developed rumen is essential for optimizing productivity and enhancing the feed-to-gain ratio in ruminants [35,36]. In our study, the rumen’s weight and proportion to the total gastric chamber weight were higher in the AL100 group compared to the AL70 and AL40 groups. This suggests that providing ample feed to growing dairy goats effectively promotes rumen development, directly impacting their DMI and growth performance. When the rumen’s relative mass stabilizes at 60% of the total stomach mass, it indicates that rumen development in goat kids has essentially reached maturity [37,38]. In this study, both the AL100 and AL70 groups, but not the AL40 group, exhibit a rumen relative mass exceeding 60% of the total stomach at the 10–20 kg and 20–30 kg Saanen dairy goats, suggesting that feeding level can substantially affect rumen maturation. Furthermore, a notable distinction was observed in this study that the impact of feeding level on rumen development is more subdued in dairy goats weighing 10–20 kg compared to their counterparts weighing 20–30 kg. This variation can be attributed to the intricate interplay between the ongoing growth trajectory of dairy goats and the concurrent evolution of their rumen development. In the 10–20 kg stage, dairy goats likely occupy an earlier stage in their growth and maturation journey with rumens that may not have fully matured, making them more responsive to changes in feed intake. In contrast, dairy goats in the 20–30 kg bracket may have entered a more advanced growth phase, with their rumens achieving a comparatively advanced state of development, making them better able to adapt to dietary changes.

### 4.4. Internal Organ Weight and Development

The growth and development of goats evolved; the quality of internal organs and organ indices reflects the functional status of the animal’s body, making it important for both theoretical research and practical production [39,40]. Previous studies reported that nutritionally restricted goat kids tend to experience decreased weight in organs such as the liver, spleen, rumen, and abomasum to sustain survival [41,42,43]. This is consistent with the results of our study, suggesting that nutrition levels play a role in influencing the growth and development of internal organs in goats [44]. Changes in the weights of visceral organs were also a manifestation of animals adapting to their nutritional status [45]. However, the effect of different feeding levels on the organ indexes of Saanen dairy goats was not significant in this study, indicating the self-regulatory mechanisms and correspondingly the harmonious growth rate of the internal organs with the overall growth of these goats. Nevertheless, a clear hierarchical structure was found in the organ development of dairy goats at two weight stages in this study. Throughout the growth and maturation process, the liver index was the highest, followed by the lung, heart, kidney, and spleen indexes. Furthermore, a noticeable trend was a descending trajectory in tandem with increasing body mass, suggesting that dairy goats commence the process of refining their organ maturation during the antecedent phase of growth. This strategic developmental disposition augments swift nutrient absorption and the optimization of the immune apparatus.

### 4.5. Allometric Growth of Tissues and Visceral Organs

Animal growth is characterized by changes in the size of visceral organs and body tissue deposition [46]. Rates of weight gain are highest from birth to puberty due to increased deposition of bone and muscle tissues [18,33]. Visceral organs play a crucial role in the energy demands of animals, highlighting the necessity for a comprehensive understanding of their growth [8]. There exists an anisotropic growth correlation between the organism’s growth rate and its bodily tissues, including bone, muscle, and fat, indicating a specific sequential growth pattern in these tissues [47]. In this study, it was found that the important tissues and organs such as muscle, visceral fat, and most of the internal organs, especially the mammary gland of dairy goats, grow faster than the body. It has been reported the fat deposition characteristics of shelter-feeding goats differ from those of grazing, with shelter-feeding goats depositing more visceral fat [30,48], which was due to the higher DMI and energy intake [30]. The growth exponent or slope (b-value) was greater at the 10–20 kg stage than at the 20–30 kg stage, mainly in muscle, skin and mohair, heart, spleen, lung, mammary gland, and abomasum. At the 20–30 kg stage, it was mainly the small intestine, kidney, rumen, omasum, large intestine, total intestine, and total GI that grew rapidly. Changes in tissue proportions were the result of different tissues developing and maturing at different rates [30]. Thus, as shelter-feeding goats in this study grow from the 10–20 kg stage to the 20–30 kg stage, a large proportion of body weight in muscle and visceral fat was observed, confirmed by the higher b value and increased ratio of meat to bone and visceral fat percentage [49]. Findings in this study elucidated that the trajectory of growth and development across various organ systems was not uniform throughout the entire growth phase spanning 10–30 kg. Understanding the growth curve of visceral organs is valuable in enhancing our comprehension of the impact of nutritional requirements on goats. This knowledge must be utilized to optimize nutritional plans effectively. The growth of organs and some tissues of dairy goats was not isochronous through the Huxley’s allometric growth equation, indicating that there is a sequential process of growth and development for different organs. Consequently, in investigating the growth and development of neonatal goats during their growth phase, it becomes imperative to acknowledge these asymmetries, enabling the tailored selection of appropriate husbandry conditions aligned with distinct weight and physiological stages, thereby enhancing the efficacy of husbandry practices. By observing the growth rate and trend of each part, we can understand in which areas animals at different growth stages show faster growth, so that we can better formulate feeding plans and management strategies.

## 5. Conclusions

From this study, it can be concluded that the growth and slaughter performance, as well as the tissue and organ development of growing Saanen dairy goats, were impaired as the feeding level fell to 70% or 40% of ad libitum intake at both 20 and 30 kg body weight stages. Additionally, the visceral fat deposition speed was substantially enhanced as the dairy goats grew from 20 to 30 kg body weight. Furthermore, in two growth stages, the muscles, visceral fat, and mammary gland of the ad libitum feeding group (AL100) displayed positive allometric growth, with their development rate being faster than that of the entire body under free-choice feeding conditions. This study provides valuable insights into the visceral organ development of Saanen goats, enhancing our understanding of their impact on nutrient requirements.

## Figures and Tables

**Table 1:** Ingredients and chemical composition of basal diets.

Ingredients, % Feeding Basis	10–20 kg	20–30 kg	Nutrients ^b^, % DM	10–20 kg	20–30 kg
Alfalfa hay	25	20	DM	87.62	86.7
Cornstalk	15	25	ME (MJ/kg)	11.02	10.6
Corn	49	45	CP	13.29	12.26
Soybean meal	8.0	7.5	EE	2.84	2.51
Dicalcium phosphate	1	0.5	NDF	29.11	33.08
Limestone	0.5	0.5	ADF	18.87	21.1
NaCl	0.5	0.5	Ash	10.91	9.62
Premix ^a^	1	1	Calcium	0.81	0.67
Total	100	100	Phosphorus	0.47	0.37

DM = dry matter; CP = crude protein; EE = Ether extract; NDF = neutral detergent fiber; ADF = acid detergent fiber; ME = Metabolizable energy (MJ/kg DM). ^a^ The premix provides the following per kg of premix: Vitamin A 600,000 IU, vitamin D3 125,000 IU, vitamin E 1000 IU, vitamin K 75 mg, vitamin B1 75 mg, vitamin B2 300 mg, vitamin B6 250 mg, vitamin B12 1 ug, D-Biotin 7.5 mg, Calcium D-pantothenate 1000 mg, folic acid 50 mg, Cu 1.0 g, Fe 3.0 g, Zn 6.0 g, Mn 2.5 g, Se 30 mg, I2 60 mg, Co 25 mg. ^b^ Nutrients for DM, CP, EE, NDF, ADF, Ash, Ca and P are measured values and ME is a calculated value (ME = GE − FE − UE − CH_4_ − E) [11].

**Table 2:** Effect of feeding level on the growth performance of female growing Saanen dairy goats.

Items	Level of Feed Intake ^1^			SEM	*p*-Value		
	AL100	AL70	AL40		Treatment	Linear	Quadratic
**10–20 kg**							
No.	8	8	8	-	-	-	-
IBW, kg	10.01	10.05	10.03	0.116	0.881	0.863	0.973
FBW, kg	20.23 ^a^	19.33 ^a^	14.04 ^b^	0.362	<0.001	<0.001	<0.001
ADG, g/d	154.31 ^a^	140.37 ^a^	59.66 ^b^	5.576	<0.001	<0.001	<0.001
DMI, g/d	769.73 ^a^	648.14 ^b^	385.85 ^c^	8.498	<0.001	<0.001	<0.001
F/G	5.70 ^b^	5.37 ^b^	7.83 ^a^	0.346	0.003	0.005	0.022
**20–30 kg**							
No.	8	8	8	-	-	-	-
IBW, kg	19.47	19.43	19.64	0.704	0.892	0.858	0.877
FBW, kg	31.28 ^a^	27.36 ^b^	21.99 ^c^	0.794	<0.001	<0.001	0.442
ADG, g/d	148.79 ^a^	103.48 ^b^	21.37 ^c^	9.231	<0.001	<0.001	0.072
DMI, g/d	1000.66 ^a^	797.06 ^b^	484.91 ^c^	23.795	<0.001	<0.001	0.260
F/G	8.40 ^b^	10.50 ^b^	19.46 ^a^	1.724	0.025	0.014	0.205

SEM = Standard error of the mean; IBW = Initial body weight; FBW = Final body weight; F/G = Feed-to-gain ratio; DMI = Average Dry Matter Intake. Within a row, means without a common letter (^a–c^) differ (*p* < 0.05). ^1^ Level of feed intake: AL100 = Ad libitum intake; AL70= 70% levels of the AL intake; AL40 = 40% levels of the AL intake.

**Table 3:** Effect of feeding level on slaughter performance of female growing Saanen dairy goats.

Item	Level of Feed Intake ^1^			SEM	*p*-Value		
	AL100	AL70	AL40		Treatment	Linear	Quadratic
**10–20 kg**							
No.	8	8	8	-	-	-	-
SBW, kg	19.74 ^a^	19.00 ^a^	13.79 ^b^	0.369	<0.001	<0.001	<0.001
EBW, kg	16.93 ^a^	15.41 ^b^	10.97 ^c^	0.290	<0.001	0.001	0.001
HCW, kg	8.66 ^a^	7.93 ^a^	5.47 ^b^	0.168	<0.001	<0.001	<0.001
Dressing percentage, %	43.53	43.34	41.92	0.655	0.203	0.106	0.456
Net meat weight, kg	5.87 ^a^	5.57 ^a^	3.46 ^b^	0.157	<0.001	<0.001	<0.001
Net meat percentage, %	29.71 ^a^	29.32 ^a^	25.13 ^b^	0.894	0.005	0.003	0.104
Carcass meat rate, %	67.78 ^a^	70.28 ^a^	63.03 ^b^	1.423	0.009	0.033	0.014
Bone weight, kg	2.79 ^a^	2.35 ^b^	2.01 ^c^	0.071	<0.001	<0.001	0.604
Meat to bone Ratio, kg/kg	2.12 ^a^	2.38 ^b^	1.77 ^c^	0.090	0.001	0.017	0.001
Visceral fat weight, g	878.09 ^a^	845.23 ^a^	488.35 ^b^	40.897	<0.001	<0.001	0.006
Visceral fat percentage, %	4.46	4.44	3.56	0.276	0.061	0.038	0.222
**20–30 kg**							
No.	8	8	8	-	-	-	-
SBW, kg	30.73 ^a^	27.06 ^b^	21.49 ^c^	0.745	<0.001	<0.001	0.279
EBW, kg	26.01 ^a^	22.54 ^b^	17.78 ^c^	0.584	<0.001	<0.001	0.365
HCW, kg	14.09 ^a^	12.06 ^b^	9.35 ^c^	0.362	<0.001	<0.001	0.426
Dressing percentage, %	45.79	44.56	43.53	0.963	0.257	0.106	0.929
Net meat weight, kg	10.36 ^a^	8.38 ^b^	6.15 ^c^	0.292	<0.001	<0.001	0.720
Net meat percentage, %	33.66 ^a^	30.97 ^b^	28.68 ^c^	0.764	0.001	<0.001	0.820
Carcass meat rate, %	73.49 ^a^	69.70 ^ab^	65.96 ^b^	1.359	0.004	0.001	0.968
Bone weight, kg	3.73	3.68	3.20	0.203	0.127	0.075	0.365
Meat to bone Ratio, kg/kg	2.77 ^a^	2.33 ^b^	1.98 ^b^	0.135	0.003	<0.001	0.769
Visceral fat weight, kg	2.94 ^a^	2.31 ^b^	1.61 ^c^	0.181	<0.001	<0.001	0.882
Visceral fat percentage, %	9.53 ^a^	8.45 ^ab^	7.46 ^b^	0.495	0.027	0.008	0.938

SEM = Standard error of the mean; SBW = Shrunk body weight; EBW = empty body weight; HCW = Hot carcass weight; AL100 = Ad libitum; AL70 = 70% AL; AL40 = 40% AL. Net meat percentage (%) = (net meat weight/SBW) × 100, Carcass meat rate (%) = (net meat weight/HCW) × 100, Visceral fat percentage (%) = (Visceral fat weight/SBW) × 100. Within a row, means without a common letter (^a–c^) differ (*p* < 0.05). ^1^ Animals of each block were slaughtered when the ad libitum kid reached 20 or 30 kg.

**Table 4:** Effect of feeding level on stomach development of female growing Saanen dairy goats.

Items	Level of Feed Intake ^1^			SEM	*p*-Value		
	AL100	AL70	AL40		Treatment	Linear	Quadratic
**10–20 kg**							
weight, g							
Rumen	382.60 ^a^	403.08 ^a^	272.50 ^b^	10.574	<0.001	<0.001	<0.001
Reticulum	67.80 ^a^	66.63 ^a^	52.22 ^b^	3.620	0.015	0.009	0.158
Omasum	74.88 ^a^	69.79 ^a^	53.69 ^b^	3.792	0.004	0.001	0.256
Abomasum	111.73 ^a^	104.18 ^a^	80.70 ^b^	5.026	0.002	0.001	0.082
Total stomach	634.64 ^a^	646.04 ^a^	459.11 ^b^	17.196	<0.001	<0.001	<0.001
weight to total stomach, %							
Rumen	60.32 ^ab^	62.43 ^a^	59.18 ^b^	0.800	0.036	0.328	0.016
Reticulum	10.67	10.30	11.49	0.573	0.352	0.330	0.286
Omasum	11.80	10.78	11.79	0.521	0.314	0.989	0.135
Abomasum	17.65	16.11	17.55	0.709	0.575	0.741	0.326
Weight to SBW, %							
Rumen	1.94 ^b^	2.12 ^a^	1.97 ^b^	0.039	0.011	0.566	0.003
Reticulum	0.34	0.35	0.38	0.020	0.344	0.185	0.559
Omasum	0.38	0.37	0.39	0.018	0.559	0.625	0.343
Abomasum	0.57	0.55	0.59	0.029	0.690	0.407	0.863
**20–30 kg**							
weight, g							
Rumen	578.07 ^a^	463.13 ^b^	354.38 ^c^	21.850	0.004	<0.001	0.902
Reticulum	103.31	90.81	77.69	4.451	0.062	<0.001	0.868
Omasum	98.14 ^a^	69.13 ^b^	62.63 ^b^	4.138	0.010	<0.001	0.032
Abomasum	131.93	123.31	115.69	5.826	0.571	0.060	0.941
Total stomach	913.73 ^a^	746.38 ^b^	610.38 ^c^	27.096	0.002	<0.001	0.618
Weight to total stomach, %							
Rumen	63.04	61.80	58.01	1.019	0.092	0.003	0.290
Reticulum	11.73	12.24	12.77	0.491	0.202	0.142	0.981
Omasum	10.71	9.35	10.28	0.538	0.651	0.565	0.082
Abomasum	14.80	16.62	18.94	0.718	0.067	<0.001	0.893
Weight to SBW, %							
Rumen	1.87	1.71	1.65	0.066	0.265	0.028	0.540
Reticulum	0.34	0.34	0.36	0.015	0.517	0.478	0.403
Omasum	0.31	0.26	0.29	0.014	0.112	0.196	0.008
Abomasum	0.44	0.46	0.54	0.022	0.169	0.003	0.310

SEM = Standard error of the mean; Total stomach weight = the weight sum of the rumen, reticulum, Omasum, and abomasum. AL100 = Ad libitum intake; AL70 = 70% levels of the AL intake; AL40 = 40% levels of the AL intake. Within a row, means without a common letter (^a–c^) differ (*p* < 0.05). ^1^ Animals of each group were slaughtered when the ad libitum kid reached 20 or 30 kg.

**Table 5:** Effect of feeding level on Organ Development of female growing Saanen dairy goats.

Internal Organs	Level of Feed Intake ^1^			SEM	*p*-Value		
	AL100	AL70	AL40		Treatment	Linear	Quadratic
**10–20 kg**							
Organ weight, g							
Heart	107.31 ^a^	102.30 ^a^	70.30 ^b^	3.748	<0.001	<0.001	0.011
Liver	443.34 ^a^	407.11 ^a^	268.96 ^b^	13.098	<0.001	<0.001	0.007
Spleen	41.60 ^a^	39.94 ^a^	25.96 ^b^	3.186	0.007	0.004	0.137
Lung	256.68 ^a^	218.21 ^b^	157.74 ^c^	11.77	<0.001	<0.001	0.458
Kidney	73.90 ^a^	65.33 ^b^	46.64 ^c^	2.504	<0.001	<0.001	0.266
Organ index							
Heart	0.55	0.54	0.51	0.020	0.273	0.230	0.750
Liver	2.25	2.15	1.95	0.087	0.294	0.028	0.692
Spleen	0.21	0.21	0.19	0.014	0.468	0.288	0.628
Lung	1.30	1.15	1.15	0.054	0.110	0.062	0.300
Kidney	0.37	0.34	0.36	0.010	0.433	0.409	0.083
**20–30 kg**							
Organ weight, g							
Heart	157.46 ^a^	128.11 ^b^	109.63 ^c^	5.620	0.020	<0.001	0.408
Liver	606.78 ^a^	483.74 ^b^	350.90 ^c^	26.858	0.008	<0.001	0.874
Spleen	59.51 ^a^	48.50 ^b^	37.37 ^c^	3.198	0.040	<0.001	0.979
Lung	299.05	246.18	247.03	16.975	0.373	0.041	0.185
Kidney	114.05	88.35	67.99	9.073	0.234	0.002	0.799
Organ index							
Heart	0.51	0.47	0.51	0.010	0.122	0.995	0.004
Liver	1.96	1.79	1.63	0.062	0.058	0.002	0.869
Spleen	0.19	0.18	0.17	0.012	0.309	0.337	0.856
Lung	0.98	0.92	1.15	0.063	1.330	0.066	0.058
Kidney	0.37	0.33	0.32	0.030	0.916	0.241	0.776

SEM = Standard error of the mean; AL100 = Ad libitum intake; AL70 = 70% levels of the AL intake; AL40 = 40% levels of the AL intake. Within a row, means without a common letter (^a–c^) differ (*p* < 0.05). ^1^ Animals of each block were slaughtered when the ad libitum kid reached 20 or 30 kg.

**Table 6:** Effects of feeding levels on tissue development of female growing Saanen dairy goats.

Internal Organs	Level of Feed Intake ^1^			SEM	*p*-Value		
	AL100	AL70	AL40		Treatment	Linear	Quadratic
**10–20 kg**							
Head	1222.50 ^a^	1162.50 ^a^	860.00 ^b^	44.233	<0.001	<0.001	0.042
Hooves	577.83 ^a^	548.06 ^a^	438.81 ^b^	16.681	<0.001	<0.001	0.072
Skin and Mohair	1231.31 ^a^	1185.91 ^a^	847.21 ^b^	34.811	<0.001	<0.001	0.004
Blood	849.19 ^a^	756.25 ^b^	518.75 ^c^	21.104	<0.001	<0.001	0.014
Small intestine	316.63 ^a^	276.31 ^a^	227.70 ^b^	15.945	0.005	0.001	0.835
Large intestine	603.75 ^a^	513.56 ^b^	399.56 ^c^	24.800	<0.001	<0.001	0.701
Uterus	19.28 ^a^	17.68 ^a^	12.41 ^b^	1.573	0.02	0.008	0.358
Mammary gland	79.69 ^a^	76.13 ^a^	39.30 ^b^	3.733	<0.001	<0.001	0.003
**20–30 kg**							
Head	1544.79 ^a^	1485.13 ^a^	1326.31 ^b^	55.767	0.025	0.010	0.492
Hooves	692.33	691.76	559.08	27.02	0.002	0.002	0.042
Skin and Mohair	1621.16 ^a^	1635.63 ^a^	1278.13 ^b^	63.069	0.023	0.002	0.037
Blood	1159.82 ^a^	970.56 ^b^	727.50 ^c^	63.451	0.028	<0.001	0.655
Small intestine	664.99 ^a^	502.56 ^b^	361.13 ^c^	37.236	0.019	0.026	0.353
Large intestine	429.97	410.44	321.19	29.147	0.132	<0.001	0.819
Uterus	42.10	26.60	25.49	4.845	0.291	0.058	0.240
Mammary gland	131.10	136.63	98.01	12.120	0.329	0.085	0.163

SEM = Standard error of the mean; AL100 = Ad libitum intake; AL70 = 70% levels of the AL intake; AL40 = 40% levels of the AL intake. Within a row, means without a common letter (^a–c^) differ (*p* < 0.05). ^1^ Animals of each block were slaughtered when the ad libitum kid reached 20 or 30 kg.

**Table 7:** Huxley’s allometric growth coefficients of tissues in female growing Saanen dairy goats.

Tissues	10–20 kg				20–30 kg			
	a	b ^†^	R^2^	*p*-Value	a	b ^†^	R^2^	*p*-Value
Muscle	−2.91	1.57 ^a^	0.834	<0.001	−2.58	1.43 ^b^	0.901	<0.001
Visceral fat	1.06	1.93 ^a^	0.669	<0.001	1.47	1.91 ^a^	0.848	<0.001
Bone	−2.05	1.02 ^b^	0.631	<0.001	−2.24	1.07 ^bc^	0.319	0.005
Skin and Mohair	3.90	1.08 ^b^	0.839	<0.001	4.26	0.94 ^c^	0.626	<0.001

a = the allometric growth constant; b = the allometric growth index; R^2^ = Percentage change in the dependent variable explained by the fitted model. ^†^ Within a column, means without a common letter (^a–c^) differ (*p* < 0.05).

**Table 8:** Huxley’s allometric growth Coefficients of organs in female growing Saanen dairy goats.

Items	10–20 kg				20–30 kg			
	a	b ^†^	R^2^	*p*-Value	a	b ^†^	R^2^	*p*-Value
Heart	0.95	1.25 ^bcde^	0.761	<0.001	1.26	1.10 ^bcd^	0.855	<0.001
Liver	1.9	1.40 ^bc^	0.786	<0.001	1.03	1.57 ^abc^	0.855	<0.001
Spleen	−1.13	1.64 ^ab^	0.631	<0.001	−1.27	1.57 ^abc^	0.615	<0.001
Lung	1.43	1.37 ^bcd^	0.665	<0.001	2.23	1.02 ^bcd^	0.16	0.06
Kidney	1.04	1.08 ^cde^	0.82	<0.001	−0.79	1.61 ^ab^	0.558	<0.001
Mammary gland	−2.17	2.21 ^a^	0.716	<0.001	−0.35	1.57 ^abc^	0.449	<0.001
Rumen	2.57	1.15 ^cde^	0.876	<0.001	1.34	1.46 ^abc^	0.802	<0.001
Reticulum	0.79	1.17 ^bcde^	0.437	<0.001	1.25	1.00 ^cd^	0.499	<0.001
Omasum	0.99	1.12 ^cde^	0.609	<0.001	−0.19	1.38 ^abc^	0.508	<0.001
Abomasum	1.28	1.16 ^bcde^	0.532	<0.001	2.14	0.82 ^d^	0.306	0.01
Total stomach	3.45	1.02 ^e^	0.907	<0.001	2.79	1.17 ^bcd^	0.826	<0.001
Small intestine	2.61	1.26 ^bcde^	0.627	<0.001	−0.14	1.94 ^a^	0.54	<0.001
Large intestine	2.08	1.23 ^bcde^	0.401	<0.001	0.85	1.56 ^abc^	0.158	0.06
Total intestine	3.31	1.17 ^bcde^	0.629	<0.001	1.6	1.59 ^abc^	0.46	<0.001
Total GI	4.21	1.05 ^de^	0.814	<0.001	3.1	1.32 ^abc^	0.657	<0.001

a = allometric growth constant; b = allometric coefficient; R^2^ = Percentage change in the dependent variable explained by the fitted model. ^†^ Within a column, means without a common letter (^a–e^) differ (*p* < 0.05).

## Data Availability

Data are contained within the article.
